# Mutant Glycyl-tRNA Synthetase (Gars) Ameliorates SOD1^G93A^ Motor Neuron Degeneration Phenotype but Has Little Affect on Loa Dynein Heavy Chain Mutant Mice

**DOI:** 10.1371/journal.pone.0006218

**Published:** 2009-07-13

**Authors:** Gareth T. Banks, Virginie Bros-Facer, Hazel P. Williams, Ruth Chia, Francesca Achilli, J. Barney Bryson, Linda Greensmith, Elizabeth M. C. Fisher

**Affiliations:** 1 Department of Neurodegenerative Disease, UCL Institute of Neurology, London, United Kingdom; 2 Sobell Department of Motor Science and Movement Disorders, UCL Institute of Neurology, London, United Kingdom; 3 MRC Centre for Neuromuscular Diseases, UCL Institute of Neurology, London, United Kingdom; Hospital Vall d'Hebron, Spain

## Abstract

**Background:**

In humans, mutations in the enzyme glycyl-tRNA synthetase (GARS) cause motor and sensory axon loss in the peripheral nervous system, and clinical phenotypes ranging from Charcot-Marie-Tooth neuropathy to a severe infantile form of spinal muscular atrophy. GARS is ubiquitously expressed and may have functions in addition to its canonical role in protein synthesis through catalyzing the addition of glycine to cognate tRNAs.

**Methodology/Principal Findings:**

We have recently described a new mouse model with a point mutation in the *Gars* gene resulting in a cysteine to arginine change at residue 201. Heterozygous *Gars^C201R/+^* mice have locomotor and sensory deficits. In an investigation of genetic mutations that lead to death of motor and sensory neurons, we have crossed the *Gars^C201R/+^* mice to two other mutants: the TgSOD1^G93A^ model of human amyotrophic lateral sclerosis and the Legs at odd angles mouse (Dync1h1^Loa^) which has a defect in the heavy chain of the dynein complex. We found the *Dync1h1^Loa/+^*;*Gars^C201R/+^* double heterozygous mice are more impaired than either parent, and this is may be an additive effect of both mutations. Surprisingly, the *Gars^C201R^* mutation significantly delayed disease onset in the *SOD1^G93A^*;*Gars^C201R/+^* double heterozygous mutant mice and increased lifespan by 29% on the genetic background investigated.

**Conclusions/Significance:**

These findings raise intriguing possibilities for the study of pathogenetic mechanisms in all three mouse mutant strains.

## Introduction

Disorders that affect motor and/or sensory neurons are relatively common and have a range of severity of symptoms, although all include muscle weakness or paralysis to variable extents. Such disorders include hereditary motor and sensory neuropathies (the Charcot-Marie-Tooth, CMT, diseases), which are the most frequent genetic disorders of the peripheral nervous system, affecting up to 1 in 2,500 people [1]. Diseases affecting solely or primarily motor neurons include motor neuron diseases (MNDs) such as amyotrophic lateral sclerosis (ALS); currently, death certificates of ∼1/400 people in England and Wales give ‘cause of death’ as forms of MND (J. Stevens pers.comm., [2]) and each year, 5000 Americans are diagnosed with ALS, 10% of whom are<40 years old [3].

Several mutant genes are known to cause sensory and/or motor neuron degeneration in humans and mice and other model organisms. In ALS, ∼10% of cases are familial (FALS), usually autosomal dominant and mutations in the ubiquitously expressed enzyme superoxide dismutase 1 (SOD1), are causal in<25% of FALS [4], [5] and in ∼3% of sporadic ALS (SALS). Mutant SOD1 takes on a toxic gain of unknown function. Several other genes with less common mutations have also been described in ALS and in the other motor neuron diseases, and mutations in these proteins have consistently implicated such cellular processes as axonal transport, RNA processing and mitochondrial function in the dysfunction of human motor and/or sensory neurons (see recent Reviews of MND and inherited neuropathy genetics [2], [6]–[12]).

With respect to axonal transport, mutations in protein subunits of the dynein-dynactin complexes, which are responsible for retrograde trafficking in axons, give rise to sensory and/or motor neuron degeneration with relatively mild symptoms and a slow onset. For example, mutations in the p150 subunit of dynactin cause a slowly progressive form of lower motor neuron disease without sensory symptoms [13].

Recently a new class of proteins, tRNA-synthetases has also been shown to be causative for motor and/or sensory neuronal dysfunction. Mutations in glycyl-tRNA synthetase (*GARS*) give rise to a range of clinical conditions generally resulting in slowly progressing muscle weakness and atrophy, with focal wasting of the musculature; the disease spectrum ranges from Charcot-Marie-Tooth type 2D (CMT2D) to severe distal spinal muscular atrophy type V (dSMA V/HMN V) [14]–[18].

Although different genetic mutations trigger disease in each disorder, it is possible that ultimately at least some of these motor/sensory neuronal diseases share certain downstream pathways that lead to neuron dysfunction and degeneration, because there may be limited numbers of ways in which neurons – particularly those with long axons - can respond to genetic or environmental insult.

We are working with a set of mouse mutants that model different types of sensory and motor neuron disease (see Table 1 for summary). We have undertaken crosses of different mutants to look for genetic interactions that may not otherwise be obvious, but which may inform us of the pathogenetic mechanisms of these disorders, which currently remain more or less unknown in every case.

**Table 1:** Phenotype summary for hemizygous *SOD1^G93A^* transgenic mice, heterozygous *Dync1h1^Loa/+^* and *Gars^C201R/^* ^+^ mouse strains.

Mouse strain	*SOD1^G93A^*	*Dync1h1^Loa^*	*Gars^C201R^*
**Type of mutation**	Transgenic overexpressor of human mutant superoxide dismutase 1 gene	ENU induced point mutation in endogenous mouse cytoplasmic dynein heavy chain 1 gene, resulting in missense mutation in protein, phenylalanine to tyrosine at residue 580	ENU induced point mutation in endogenous mouse glycyl-RNA synthetase gene, resulting in missense mutation in protein, cysteine to arginine at residue 201
**Primary reference**	[19]	[22]	[25]
**Genetic data**	Autosomal dominant trait; transgene inserted into mouse chromosome 12 [27]	Autosomal dominant mutation on mouse chromosome 12	Autosomal dominant mutation on mouse chromosome 6
**Human disease model**	Amyotrophic lateral sclerosis	None described	Charcot-Marie-Tooth type 2D (some features)
**Breeding**	Females are infertile; equal numbers of male and female progeny are produced	Males and females are fully fertile; equal numbers of male and female progeny are produced	Males and females are fully fertile; equal numbers of male and female progeny are produced
**Lifespan**	∼130 days for humane endpoint, depending on genetic background	Over 2 years	Over 2 years
**Age at onset**	∼90 days depending on genetic background	1–3 months depending on genetic background	At least 1 month depending on genetic background
**Symptoms at onset**	Paralysis and weight loss	Limb clasping when suspended by the tail; low based gait in some mice	Mild deficits in grip strength and fine motor control
**Nerve and muscle**	Motor neuron degeneration, loss of muscle force, muscle atrophy	Mild motor neuron loss; pronounced loss of prioprioceptors, some loss of muscle force, no obvious muscle pathology so far	Reduction in axon diameter of peripheral nerves, alteration in sensory nerve conduction, neuromuscular junction deficits; some loss of muscle force, changes in muscle fiber type
**Mouse crosses**	Crossed to *Dync1h1^Loa^* [26]; crossed to *Gars^C201R^* this paper	Crossed to *Gars^C201R^* this paper; crossed to *SOD1^G93A^* [26]	Crossed to *SOD1^G93A^* this paper; crossed to *Dync1h1^Loa^* this paper

Note that the phenotype of homozygous *Dync1h1^Loa/+^* and *Gars^C201R/^* ^+^ mice is considerably more severe and few homozygous animals of either strain survive much beyond birth. Mice with two copies of the *SOD1^G93A^* transgene array are not viable.

The first mouse we are working with, the TgSOD1^G93A^ transgenic (SOD1^G93A^), models human amyotrophic lateral sclerosis (ALS) [19]. This mouse is widely used and carries a human mutant transgene array with a glycine to alanine mutation at residue 93 of SOD1, which is causative for ALS in humans, and which results in a similar phenotype in the mice who succumb to endstage disease at ∼130 d of age (depending on genetic background)[19].

The second mouse we are characterizing is the Legs at odd angles mouse (Dync1h1^Loa^) which does not model a specific human disease, but the mice are of interest because heterozygotes have a mild motor deficit and a pronounced loss of proprioceptive neurons arising from a point mutation in the heavy chain gene (*Dync1h1*) of the cytoplasmic dynein complex [20]–[24].

The third mouse of interest is a new model we have recently described [25], which carries a point mutation leading to a non-conservative amino acid change in the glycine t-RNA synthetase gene (*Gars*). The new mouse model, *Gars^C201R^*, has a cysteine to arginine change at position 201, which, in heterozygous animals, results in deficits in grip strength, decreased motor flexibility, disruption of fine motor control, as well as a reduction in axon diameter in peripheral nerves and alterations in nerve conduction with neuromuscular junction deficits; this phenotype is variable depending on genetic background [25].

*SOD1*, *Dync1h and Gars* are ubiquitously expressed genes and their protein products are essential for all cell types. However, mutations in these genes specifically affect sensory and/or motor neuron function. The mechanistic links between aberrant protein and pathological mechanism, and cell type specificity, remain unclear for all three mutations. As part of an investigation of our mouse models, we have undertaken a classic genetic approach: crossing the mutants in different combinations to determine if we can detect an interaction between these proteins, which may lead us to new pathways of pathogenesis. We and others have already reported on the results of a *Dync1h1^Loa/+^* x *SOD1^G93A^* cross: the double heterozygous progeny, *Dync1h1^Loa/+^*;*SOD1^G93A^* have an intriguing delayed disease onset compared to their *SOD1^G93A^* hemizygous littermates and parents, and an extended lifespan reported to be between 9% and 28% [20], [21], [23], [26]. Here we report our results from assessing the progeny of the two other possible crosses: *Gars^C201R/+^* x *SOD1^G93A^* and *Gars^C201R/+^* x *Dync1h1^Loa/+^*. We find *Dync1h1^Loa/+^*;*Gars^C201R/+^* double heterozygote progeny have a more severe phenotype than their mildly affected parents, although this may simply be a manifestation of additive effects. However, in contrast *SOD1^G93A^*;*Gars^C201R/+^* double heterozygotes have an extension of lifespan and preservation of motor neurons which is similar to that of *Dync1h1^Loa/+^*;*SOD1^G93A^* double heterozygotes. The results of these mouse crosses may help inform us further of novel protein interactions and therefore the mechanism of both *SOD1^G93A^* toxicity, and the effect of the *Gars^C201R^* mutation.

## Results

### Analysis of double heterozygous progeny from a *SOD1^G93A^* x *Gars^C201R/^* ^+^ cross

The *SOD1^G93A^* males for this cross were on an SJL x C57BL/6 background (see Materials and Methods). Onset of motor neuron disease symptoms (determined by start of weight loss) typically begins at ∼110 days in this colony with mice reaching disease endpoint (defined as loss of righting reflex for 20 secs or weight loss of 15%) at ∼130 days. The *Gars^C201R/^* ^+^ females for this cross came from a colony in which the mutation is maintained by backcrossing to C57BL/6J mice. *SOD1^G93A^* hemizygous females are infertile hence we could not carry out the reciprocal *SOD1^G93A^* female x *Gars^C201R/^* ^+^ male cross.

*Gars^C201R/^* ^+^ heterozygous females (n = 14, N1-4 on the C57BL/6 background) were crossed with *SOD1^G93A^* males to produce the expected four genetically distinct groups of littermates: wildtype 26 males and 22 females (total 48); *SOD1^G93A^* 19 males and 19 females (total 38); *Gars^C201R/+^* 15 males and 21 females (total 36); *SOD1^G93A^*;*Gars^C201R/+^* 15 males and 18 females (total 33). Thus there was some deviation from the expected Mendelian ratio of 25% per genotype although this difference was not statistically significant; there was no gender bias. The *Gars^C201R^* mutation lies on mouse chromosome 6 [25] and the *SOD1^G93A^* transgene array lies on mouse chromosome 12 [27], thus the two loci are segregating independently.

### Extended lifespan in *SOD1^G93A^*;*Gars^C201R/+^* double heterozygotes

**Figure 1:**
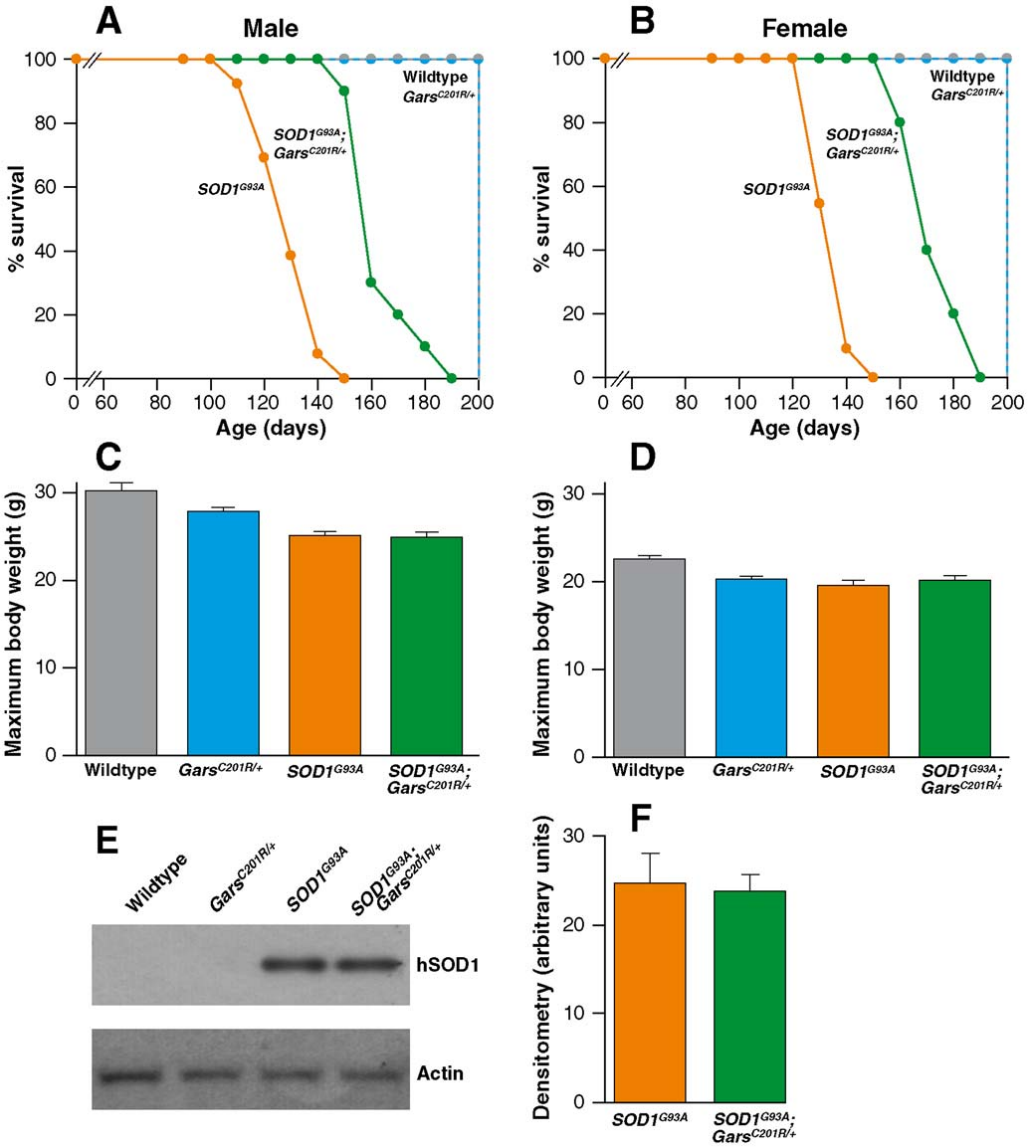
Lifespan of littermates from the *SOD1^G93A^* x *Gars^C201R/^* ^+^ cross. (A) Male mice: *SOD1^G93A^* n = 13; *SOD1^G93A^*;*Gars^C201R/+^* n = 10. (B) Female mice: *SOD1^G93A^* n = 11; *SOD1^G93A^*;*Gars^C201R/+^* n = 10. Maximum body weights. Weights were recorded between 90 and 110 days which corresponds to the maximum body weight prior to weight loss as the disease progresses. (C) Males: wildtype 30.2±0.9 g (n = 13); *SOD1^G93A^* 25.1±0.4 g (n = 13); *Gars^C201R/+^* 27.9±0.4 g (n = 12); *SOD1^G93A^*;*Gars^C201R/+^* 24.9±0.6 g (n = 11). (D) Females: wildtype 22.6±0.4 g (n = 13); *SOD1^G93A^* 19.5±0.6 g (n = 12); *Gars^C201R/+^* 20.3±0.3 g (n = 9); *SOD1^G93A^*;*Gars^C201R/+^* 20.2±0.5 g (n = 11). (E) A representative western blot of human and mouse SOD1 using spinal cord homogenate from 4 month old mice; hSOD is human SOD1 protein. (F) Quantification of the human SOD1 protein levels in shows no transgene loss in *SOD1^G93A^*;*Gars^C201R/+^* compared to *SOD1^G93A^* mice mice (n = 4 per cohort).

We examined whether the mutation in glycine tRNA synthetase inherited from the *Gars^C201R/+^* parents, altered the lifespan of *SOD1^G93A^* mice by comparing all four genotypes of progeny mice (Figure 1A,B). Wildtype and *Gars^C201R/+^* mice had a normal lifespan and *SOD1^G93A^* progeny a significantly reduced lifespan of 126±3 days for males (n = 13) and 131±2 days females (n = 11), with disease end-stage defined as above. Surprisingly, *SOD1^G93A^*;*Gars^C201R/+^* double heterozygote males lived for 163±4 days (n = 10) and females lived for 170±3 days (n = 10). These mice had an increase of lifespan of 37 days for males and 39 days for females compared to *SOD1^G93A^* littermates, averaging to approximately 29% for both sexes.

Body weight was assessed weekly and as can be seen in Figure 1C,D, the maximum body weights of all three mutant genotypes were lighter than their wildtype littermates and male mice were generally 20% heavier than their female counterparts, on the genetic backgrounds studied.

The *SOD1^G93A^* transgene array is known to delete occasionally, which results in an extension of lifespan of the mice because the severity of the phenotype depends on the level of mutant SOD1 protein expression. While it is extremely unlikely that the extension of lifespan seen in all 20 *SOD1^G93A^*;*Gars^C201R/+^* double heterozygotes was caused by deletion in the transgene array in mice with this genotype only, nevertheless we quantified the level of SOD1 protein expression in the four progeny genotypes. Spinal cords from 4 month old wildtype, *SOD1^G93A^* hemizygote, *Gars^C201R/^* ^+^ heterozygote and *SOD1^G93A^*;*Gars^C201R/+^* double heterozygotes were homogenized and western blots of these homogenates were probed with Novocastra NCL-SOD1 antibody, which detects both human and mouse SOD1 [26]. The membrane was reprobed with anti β−actin as an internal protein loading control and signals were quantified as described in Materials and Methods. We found human SOD1 was present in *SOD1^G93A^* hemizygotes, and *SOD1^G93A^*;*Gars^C201R/+^* double heterozygotes only (Figure 1E). When the blots were quantified we found no significant differences in human SOD1 protein levels between *SOD1^G93A^* hemizygotes (24.7±3.3 arbitrary units), and *SOD1^G93A^*;*Gars^C201R/+^* double heterozygotes (23.8±1.9 arbitrary units) (Figure 1F), indicating the extended lifespan of double heterozygotes was not the result of a deletion in the *SOD1^G93A^* transgene array (n = 4 for all cohorts).

### Disease characteristics in *SOD1^G93A^*;*Gars^C201R/+^* progeny

Disease phenotype and progression in each of the 4 littermate cohorts were also examined by *in vivo* physiological analysis of the hindlimb muscles, tibialis anterior (TA) and extensor digitorum longus (EDL) in 120 d old female mice. For each genotype cohort, n = 5 unless otherwise stated.

**Figure 2:**
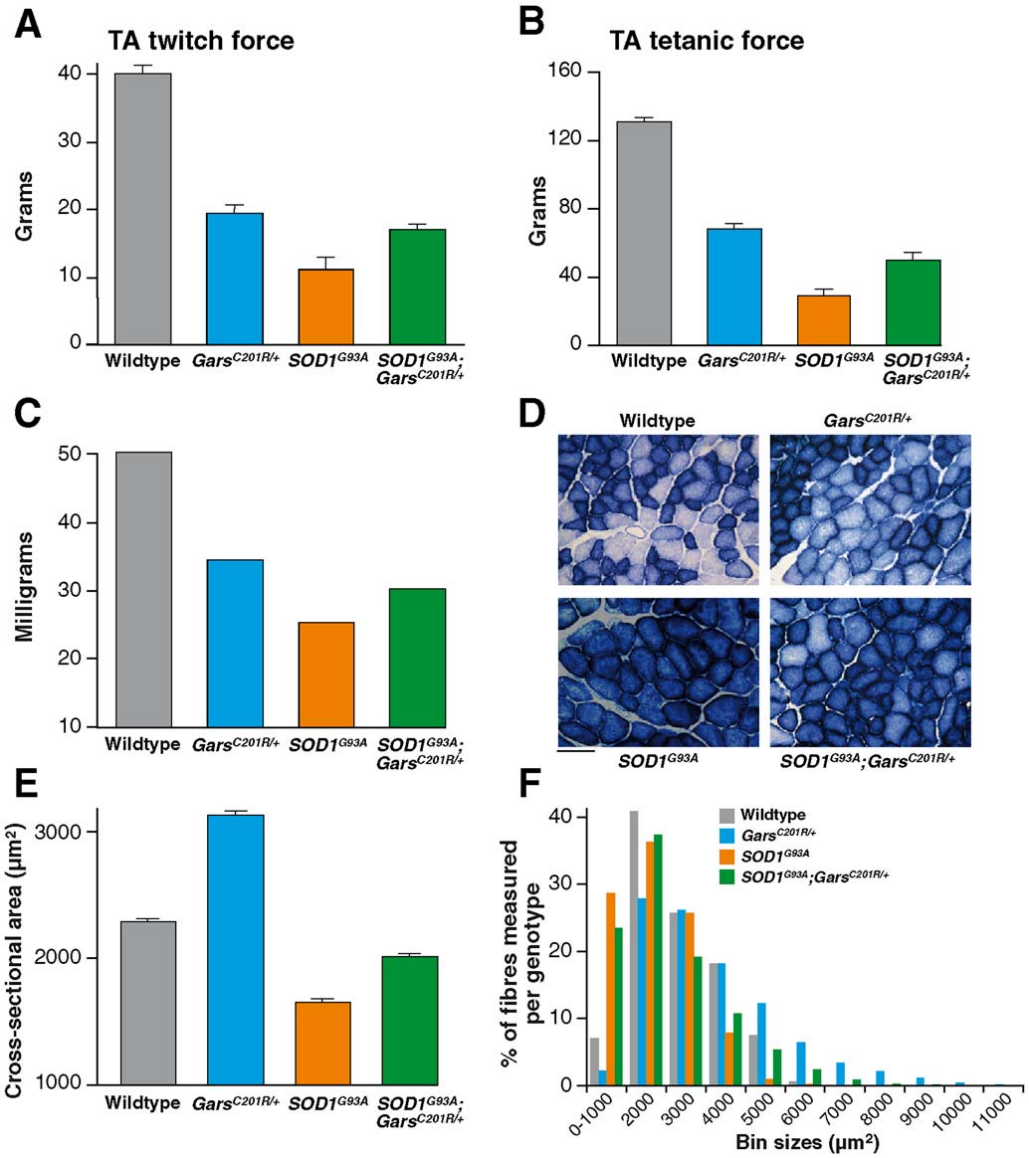
Muscle force and phenotype in TA muscles from littermates of the *SOD1^G93A^* x *Gars^C201R/^* ^+^ cross at 120 days of age. The bar charts show: (A) the maximum twitch and, (B) tetanic force generated by TA muscles and, (C) the mean weight of TA muscles from each genotype. (D) shows examples of TA muscle sections stained for succinate dehydrogenase (SDH), an indicator of oxidative capacity. Scale bar = 70 µm. (E) From sections such as these the cross sectional area (CSA) of TA muscle fibers in SDH stained sections was assessed and the mean CSA is shown in the bar chart (n = 3 per genotype). (F) The distribution of muscle fiber area is shown in the frequency distribution histogram (n = 5 per genotype). Errors bars represent SEM (not visable in C).

Both twitch and tetanic muscle force were assessed. As can be seen in Figures 2A and B, TA muscles in *SOD1^G93A^* mice were significantly weaker than the corresponding muscles in their wildtype littermates. Whereas TA muscles in wildtype mice had a maximum twitch and tetanic force of 40.2±1.2 g. and 131.5±2.3 g respectively, in *SOD1^G93A^* littermates, twitch force was reduced to only 11.2±1.6 g (P≤0.001) and tetanic force was only 28.6±4.0 g (P = 0.009).

In *Gars^C201R/+^* mice, there was also a reduction in force of TA muscles, so that twitch and tetanic tension was 19.5±1.2 g and 68.0±3.7 g, respectively, which is significantly less than that of their wildtype littermates (P≤0.001). In *SOD1^G93A^*;*Gars^C201R/+^* double heterozygotes, the comparable TA maximum twitch force was 16.8±1.1 g, and the maximum tetanic force was 49.9±4.1 g, which is significantly weaker than the corresponding values in wildtype mice (P = 0.037 and P = 0.009 respectively). However, although the TA muscles in the double heterozygote, *SOD1^G93A^*;*Gars^C201R/+^* mice were markedly weaker than wildtype littermates, they were significantly stronger than TA muscles in *SOD1^G93A^* mice (i.e. twitch force: 16.8±1.1 g compared to 11.2±1.6 g (P≤0.001); tetanic force: 49.9±4.1 g compared to 28.6±4.0 g (P = 0.009)).

The TA muscles from all 4 genotypes were removed and weighed at the end of the physiological tests. The weakness observed in TA muscles of *SOD1^G93A^*, *Gars^C201R^* ^/+^ and *SOD1^G93A^*;*Gars^C201R/+^* double heterozygotes was reflected in a significant reduction in the weight of these muscles compared to those of wildtype littermates (Figure 2C; P≤0.001).

We also examined the force characteristics of the EDL muscles in each genotype cohort. As observed in TA, EDL muscles of *SOD1^G93A^* and *Gars^C201R^* mice were significantly weaker than EDL in wildtype littermates (see Figure S1A and B, Supplementary Table S1). In contrast to the findings for TA, there was no significant difference (i.e. improvement) in the force output of EDL in *SOD1^G93A^*;*Gars^C201R/+^* double heterozygotes compared to their *SOD1^G93A^* littermates. Further analysis of the contraction characteristics of EDL in the *SOD1^G93A^*;*Gars^C201R/+^* double heterozygotes also established that there was no difference in these characteristics from those in EDL of *SOD1^G93A^* or *Gars^C201R/+^* heterozygotes, so that time to peak, half relaxation time and the fatigue index of EDL muscles were similar in all genotype cohorts (Supplementary Table S2).

**Figure 3:**
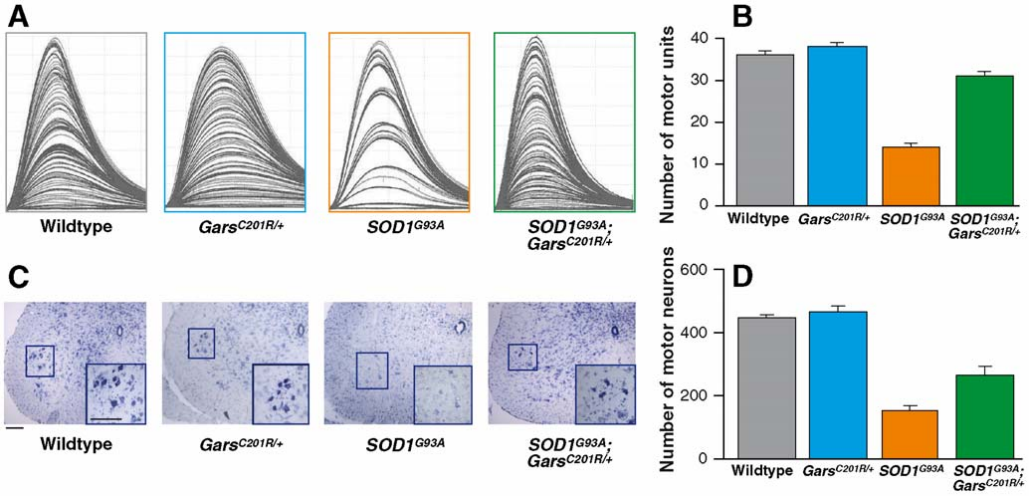
Motor unit and motor neuron survival in littermates from the *SOD1^G93A^* x *Gars^C201R/^* ^+^ cross at 120 days of age. (A) shows examples of motor unit traces from EDL muscles of mice of each genotype. (B) The bar chart shows the mean motor unit survival in each littermate cohort (n = 5 female littermates per genotype at 120 days of age). (C) Examples of cross sections of spinal cord from mice of each genotype showing motor neurons within the ventral horn are shown. The sciatic motor pools are identified within the magnified inserts. Scale bars = 100 µm.The bar chart in (D) shows the mean motor neuron survival (n = 3 per genotype). Error bars represent SEM.

We also assessed the number of motor units that innervated the hindlimb muscles of mice of each genotype. TA is normally innervated by a very large number of motor units so that a reliable assessment of motor unit number in TA is not possible using the method of motor unit (MU) estimation employed in this study. Therefore, despite the absence of significant differences in EDL muscle force characteristics and since motor unit changes are not always immediately reflected in alterations in muscle phenotype, we assessed the number of functional motor units that innervated EDL muscles of mice of each genotype. Typical examples of motor unit traces from EDL muscles of mice of each genotype are shown in Figure 3A, and the mean number of surviving motor units in each experimental group (n = 5 for each genotype) is summarized in the bar chart (Figure 3B). In EDL muscles of *SOD1^G93A^* mice, there is a significant reduction in the number of surviving motor units at 120 d compared to wildtype littermates. Thus, in wildtype mice, EDL is innervated by 36±1 motor units but in *SOD1^G93A^* mice, only 14±1 motor units survive. There is no loss of motor units in *Gars^C201R/+^* mice, and 38±1 motor units survive. However, in *SOD1^G93A^*;*Gars^C201R/+^* double heterozygotes, there was a surprising and significant increase in motor unit survival compared to *SOD1^G93A^* mice, so that 31±1 motor units survive (P≤0.001).

Following completion of the in vivo physiological analysis of muscle function and motor unit survival, the TA muscles and spinal cords of each animal were removed for histological analysis. TA muscle is normally a fast-contracting muscle that fatigues rapidly when repeatedly stimulated. In *SOD1^G93A^* mice this characteristic feature of TA changes dramatically and by 120 days of age TA becomes a slow, fatigue-resistant muscle. These changes in the fatigue characteristics of TA muscles of *SOD1^G93A^* mice are reflected in alterations in the histochemical properties of the muscle fibers, which show an increase in oxidative capacity, staining darkly for the oxidative enzyme succinate dehydrogenase (SDH). We stained TA muscles for SDH activity and compared the pattern of staining in wildtype mice to that observed in TA muscles of *SOD1^G93A^*, *Gars^C201R^* ^/+^ and *SOD1^G93A^*;*Gars^C201R/+^* double heterozygote mice. As can be seen in Figure 2D, there is a dramatic increase in the number of darkly stained fibers in TA muscles of *SOD1^G93^* ^A^ mice, and a slight increase in the proportion of dark fibers in TA of *Gars^C201R^* ^/+^ mice. However, in *SOD1^G93A^*;*Gars^C201R/+^* double heterozygotes there was a greater proportion of lightly stained fibers than present in *SOD1^G93A^* muscles, although significantly more fibers were darkly stained than in TA muscles of wildtype or *Gars^C201R^* ^/+^ mice.

It can also be seen in Figure 2D that the size of the muscle fibers appears to vary among the different genotypes. We therefore assessed the cross-sectional area (CSA) of TA muscle fibers in SDH stained sections from mice of each genotype. The bar chart in Figure 2E summarizes the results and shows that the mean CSA of TA muscle fibers of wildtype mice is 2292±14 µm^2^ (n = 3), and in *SOD1^G93A^* mice this is reduced to 1648±20 µm^2^ (n = 3). In contrast, there is a significant increase in the mean CSA of TA muscle fibers in *Gars^C201R^* ^/+^ mice to 3130±28 µm^2^ (n = 3). In TA muscles of *Gars^C201R/+^*;*SOD1^G93A^* double heterozygotes, the mean CSA is 2013±14 µm^2^ (n = 3). Thus there is a significant decrease in the CSA of TA muscle fibers in *SOD1^G93A^* mice and a significant increase in *Gars^C201R^* ^/+^ mice compared to controls (P<0.001). However, in the *Gars^C201R/+^*;*SOD1^G93A^* mice the mean CSA of TA muscle fibers is greater than that in *SOD1^G93A^* mice, and is similar to that observed in wildtype animals. In order to distinguish whether these findings of mean CSA were due to a loss or gain of a specific group of muscle fibers, we undertook a morphometric analysis of the muscle fiber size distribution for each genotype (Figure 2F). Our results show that in *SOD1^G93A^* mice, there are fewer large muscle fibers present than in the wildtype TA muscle. In *Gars^C201R/+^* mice, there is a greater proportion of large fibers present, and some of the fibers have a larger CSA observed in wildtype muscles. However, in *SOD1^G93A^*;*Gars^C201R/+^* mice, the TA muscles have a greater proportion of large fibers than observed in *SOD1^G93A^* mice, suggesting that the presence of the *Gars^C201R/+^* mutation has prevented the reduction in muscle fiber area that otherwise occurs in TA muscles of *SOD1^G93A^* mice by 120 days.

It is well established that the loss of muscle force and reduction in motor unit survival in 120 d *SOD1^G93A^* mice is due to the degeneration of a large number of motor neurons in the lumbar spinal cord. To determine if the improvements in muscle function and morphology in *SOD1^G93A^*;*Gars^C201R/+^* were reflected in an increase in motor neuron survival in these mice compared to their *SOD1^G93A^* littermates, motor neuron survival was assessed in littermates of each genotype. Examples of sections of lumbar spinal cords from each experimental group are shown in Figure 3C and the mean motor neuron survival is summarized in the bar chart (Figure 3D; n = 3 per genotype). As expected, in *SOD1^G93A^* mice there is a dramatic decrease in the number of motor neurons that survive by 120 d and in the portion of the sciatic motor pool examined (see methods for details) only 155±10 motor neurons survive compared to 447±9 motor neurons in the same portion of the sciatic motor pool in wildtype littermates (P<0.001). However, in *SOD1^G93A^*;*Gars^C201R/+^* littermates there is a significant increase in motor neuron survival, and 263±28 motor neurons survive, which is significantly greater than in *SOD1^G93A^* littermates (P = 0.023). In *Gars^C201R/+^* mice there is no loss of motor neurons, at least at this stage and 467±11 motor neurons are present in the sciatic motor pool at 120 d.

### Investigating protein levels and interactions in the a *SOD1^G93A^* x *Gars^C201R/^* ^+^ cross

Our previous studies have shown that the levels of GARS protein are significantly increased in *Gars^C201R/+^* heterozygotes compared to wildtype littermates at 15 days of age, but there is no significant difference in GARS levels in brain by 90 days of age [25]. To quantify GARS protein levels in the progeny of this cross, spinal cord protein homogenates were made from wildtype, *SOD1^G93A^*, *Gars^C201R/+^* and *SOD1^G93A^*;*Gars^C201R/+^* littermates at 120 days of age (n = 4 for all genotypes). GARS levels were normalized to β−actin (an internal protein loading control) as described previously [25]. We found no significant difference in normalised GARS protein levels between wildtype (10.7±1.4 arbitrary units) and *SOD1^G93A^* (9.4±1.5 arbitrary units) animals. However the GARS levels in *Gars^C201R/+^* mice (18.5±1.9 arbitrary units) and in *SOD1^G93A^*;*Gars^C201R/+^* mice (19.2±2.4 arbitrary units) were both significantly higher than that of wildtype animals (p = 0.034 compared to *Gars^C201R/+^* and p = 0.047 compared to *SOD1^G93A^*;*Gars^C201R/+^*, see Supplementary Figure S2A, B). This difference in protein levels compared to that found in [25] may reflect the mixed genetic background segregating in the current cross. There was no significant difference in GARS levels between *Gars^C201R/+^* and *SOD1^G93A^*;*Gars^C201R/+^* mice.

Other studies have demonstrated that lysyl-tRNA synthetase (KARS) can interact with SOD1 and this interaction is stronger for mutant than wildtype SOD1 [28], [29]. Thus we investigated if we could detect an interaction between GARS and SOD1 in our mice, by co-immunoprecipitation studies with all four genotypes. Immunoprecipitation was performed upon native spinal cord homogenates from 120 day old mice (n = 3 per cohort) using either SOD1 or GARS antibodies linked to agarose beads to pull down the protein of interest and any interacting proteins. The pull downs were then probed for interacting proteins on western blots. In these studies we could find no evidence of a GARS-SOD1 protein interaction in any of the four genotypes studied (data not shown).

### Analysis of double heterozygous progeny from a *Dync1h1^Loa/+^* x *Gars^C201R/^* ^+^ cross

*Dync1h1^Loa/+^* mice are maintained by backcrossing to C57BL/6J mice and all mice used as parents in this cross were at least generation N10 and are thus congenic for this background. The *Gars^C201R/^* ^+^ parents used in this cross were as described above i.e. maintained by crossing to C57BL/6 but not congenic. The *Dync1h1^Loa/+^* mutation leads to defects in motor and sensory neurons, but in heterozygotes the phenotype is mild and there are no known defects in lifespan or fertility [22].

*Dync1h1^Loa/+^* heterozygous mice (n = 4 males) were crossed with *Gars^C201R/^* ^+^ mice (n = 8 females, N1-4 from the C57BL/6 backcross), producing the four expected genotypes of progeny: wildtype 25 males and 23 females (total 48); *Dync1h1^Loa/+^* 11 males and 11 females (total 22); *Gars^C201R/+^* 37 males and 24 females (total 61); *Dync1h1^Loa/+^*;*Gars^C201R/+^* 21 males and 12 females (total 33). Thus there was some deviation from the expected Mendelian ratio of 25% per genotype in that we see a loss of animals carrying the *Dync1h1^Loa^* mutation, which is in accordance with our previous findings for this mutation [22], [26]. All mice were genotyped for the *Gars^C201R^* mutation and *Dync1h1^Loa^* mutation as previously described [22], [25], [26] at approximately 28 days of age. All phenotypic characterization took place blind to genotype which was then decoded. The dynein heavy chain gene lies on Mmu12 and thus segregates independently of the *Gars* locus.

### Characteristics of *Dync1h1^Loa/+^*;*Gars^C201R/+^* progeny

**Figure 4:**
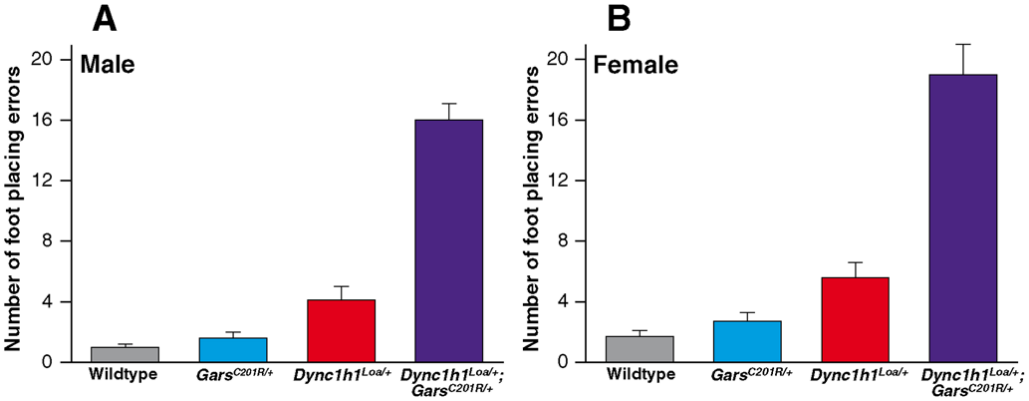
Wirewalk footplacing errors in progeny from the *Dync1h1^Loa/+^* x *Gars^C201R/^* ^+^ cross at 7 months of age. Mean average numbers of footplacing errors for each genotype. (A) Males: wildtype n = 12, *Dync1h1^Loa/+^* n = 9, *Gars^C201R/+^* n = 12, *Dync1h1^Loa/+^*;*Gars^C201R/+^* n = 12. (B) Females: wildtype n = 11, *Dync1h1^Loa/+^* n = 11, *Gars^C201R/+^* n = 10, *Dync1h1^Loa/+^*;*Gars^C201R/+^* n = 9. Error bars show SEM.

Mice from all four genotypes were tested for grip strength at 7 months of age and no significant difference was detected between the *Gars^C201R/+^* heterozygotes and sex-matched *Dync1h1^Loa/+^*;*Gars^C201R/+^* double heterozygotes (Supplementary Figure S3A, B). However, starting at 4 months of age, a gait deficit was observed that was more pronounced in the *Dync1h1^Loa/+^*;*Gars^C201R/+^* double heterozygous animals compared to their littermates and parents (Movie S1, Movie S2, Movie S3, Movie S4). A wire walk test was carried out at 7 months of age to quantify this defect: in this test mice are put on a mesh cage lid and timed as they walk for one minute on the horizontal lid while the number of ‘foot placing errors’ - occasions in which the mouse misses the wire mesh and puts a forepaw or hindpaw into the hole between the wires - is counted. The double heterozygotes performed significantly worse than their wildtype, *Dync1h1^Loa/+^* or *Gars^C201R/+^* littermates (Figure 4). For male progeny, *Dync1h1^Loa/+^*;*Gars^C201R/+^* double heterozygotes made 16±1 foot placing errors (n = 12) compared to their littermates (wildtype 1±0 foot placing errors, n = 12; *Dync1h1^Loa/+^* 4±1 foot placing errors, n = 9; *Gars^C201R/+^* 2±0 foot placing errors, n = 12). Female *Dync1h1^Loa/+^*;*Gars^C201R/+^* double heterozygotes made 19±2 foot placing errors (n = 9) compared to their littermates (wildtype 2±0 foot placing errors, n = 11; *Dync1h1^Loa/+^* 6±1 foot placing errors, n = 11; *Gars^C201R/+^* 3±1 foot placing errors, n = 10). This novel gait deficit may represent the additive effects of both mutations, rather than any interaction between them. We also found that by 7 months of age the *Dync1h1^Loa/+^*;*Gars^C201R/+^* double heterozygotes had developed tremor – 5 out of 5 mice studied showed continuous whole body tremors when observed on the wire mesh of a cage lid; tremors were not seen in wildtype littermates.

In vivo physiological assessment of TA and EDL muscle force was also performed in 9 months old animals from the *Dync1h1^Loa/+^* x *Gars^C201R/^* ^+^ cross (all females, n = 3 for all genotypes except for *Dync1h1^Loa/+^* where n = 2, Supplementary Table S3; Supplementary Figure. S4). Wildtype littermates had a maximum tetanic force in TA muscle of 126.2±2.7 g. As in the experiments described above, TA muscles in the *Gars^C201R^* ^/+^ mice were significantly weaker and had a maximum tetanic force of 60.6±3.0 g (P = 0.002). In the double heterozygote *Dync1h1^Loa/+^*;*Gars^C201R/+^* mice, the maximum force was 52.6±7.3 g, which is not significantly different from that observed in their *Gars^C201R^* ^***/*+**^ littermates (P = 0.004). The *Dync1h1^Loa/+^* mice were slightly weaker than their wildtype littermates, but to a lesser extent than *Gars^C201R^* ^/+^, so that the maximum tetanic force of TA muscles in *Dync1h1^Loa/+^* mice was 109.0±5.1 g (P = 0.01). This finding confirms our previous results that also show that there is a small reduction in the force of hindlimb muscles in *Dync1h1^Loa/+^* mice (Kieran et al, 2005). We also assessed muscle force in EDL muscles of mice of each genotype, and found no significant difference in the maximum muscle force or the muscle contractile characteristics of EDL muscles from the 4 genotypes of the *Dync1h1^Loa/+^* x *Gars^C201R/^* ^+^ cross (Supplementary Table S3 and Supplementary Fig. S4).

The number of motor units innervating the EDL muscles in mice of each genotype was also examined and the results showed that there was no significant difference in motor unit number between any of the experimental groups (see Supplementary Figure S4). Together, these results indicate that the *Dync1h1^Loa/+^* mutation does not have an additional effect on the muscle force of the *Gars^C201R^* ^***/*+**^ mice since both TA and EDL muscles from the *Gars^C201R^* ^***/*+**^ mice and the double heterozygote *Dync1h1^Loa/+^*;*Gars^C201R/+^* mice pull similar muscle forces and have a normal complement of motor units.

### Investigating protein levels and interactions in the *Dync1h1^Loa/+^* x *Gars^C201R/^* ^+^ cross

Spinal cord homogenates of 4 month old mice (n = 4 for all four genotypes) were assessed for GARS protein levels by quantitative western blot (normalizing to β actin, as described above). We found no significant difference in GARS protein levels between wildtype and *Dync1h1^Loa/+^* animals (15.0±1.9 and 15.8±2.9 arbitrary units for wildtype and *Dync1h1^Loa/+^* respectively). However the GARS levels in *Gars^C201R/+^* animals (33.1±1.2 arbitrary units) and in *Dync1h1^Loa/+^*;*Gars^C201R/+^* animals (27.0±1.2 arbitrary units) were both significantly higher than that of wildtype animals (p = 0.002 compared to *Gars^C201R/+^* and p = 0.009 compared to *Dync1h1^Loa/+^*;*Gars^C201R/+^*) (see Supplementary Figure S2C,D).

We investigated whether there was any direct interaction between the dynein complex (which, in its native form, would include the dynein heavy chain) and GARS by co-immunoprecipitation studies. Immunoprecipitation was performed upon native spinal cord homogenates of 4 month old mice (n = 4 for all four cohorts) using either dynein intermediate chain or GARS antibodies linked to agarose beads to pull down the protein of interest and any interacting proteins as described above. The pull downs were then probed for interacting proteins on western blots. We could find no evidence of a dynein/GARS protein interaction in any of the four genotypes studied. In all four genotypes the dynein intermediate chain immunoprecipitation pulled down the dynein light chain LC8. This suggests that the dynein complex itself is intact in all four genotypes (data not shown).

## Discussion

The *Gars^C201R^* mouse is a valuable new resource both for studying the pathogenesis of defects in this enzyme and for use as a genetic tool to uncover interactions with cellular pathways affecting motor and/or sensory neuronal function. We have taken a standard genetics approach to cross this mouse strain with two other strains that carry mutations either modelling human neurodegenerative disease such as ALS, (*SOD1^G93A^* transgenics) or known to have deficits that affect the motor/sensory system (*Dync1h1^Loa/+^* mice).

In analyzing the results of these crosses we unexpectedly found an amelioration of the motor neuron disease phenotype caused by the *SOD1^G93A^* transgene array in the presence of the *Gars^C201R/^* ^+^ genotype. We and others, have previously observed a similar effect in *Dync1h1^Loa/+^*;*SOD1^G93A^* double heterozygotes although this finding has not yet been explained at a molecular level [20], [21], [23]. Another dynein heavy chain mutant allele, *Dync1h1^Cra1/+^*, also extends *SOD1^G93A^* mouse lifespan [30] while a third allele, *Dync1h1^Swl/+^* does not [21].

We believe the *Gars^C201R^* allele, as opposed to any other gene, is having an effect on the *SOD1^G93A^* phenotype because (1) lifespan is consistent between *SOD1^G93A^* hemizygous parents and progeny, (2) the increase of lifespan only occurs in *SOD1^G93A^* hemizygotes that also carry the *Gars^C201R^* mutation (double heterozygotes). Thus lengthening of lifespan is consistent with *Gars^C201R^* genotype, and not genetic background effects (although it is formally possible but highly unlikely the effect is due to a BALB/c wildtype gene closely linked to the *Gars* locus, as the original mutation was produced on a BALB/c background [25]). We note the lifespan of *SOD1^G93A^* hemizygotes is extended when they are bred onto a C57BL/6 background (our data and [31], [32]) but again, if the lifespan extension were due to the random segregation of C57BL/6 alleles from the genetic background of the non-congenic *Gars^C201R/+^* parents, we would see the same extension in *SOD1^G93A^* hemizygotes as well as in the double heterozygotes, and this is not the case.

We did not detect any direct GARS-SOD1 protein interactions, and therefore have to assume that the effect of the *Gars^C201R^* on the *SOD1^G93A^* phenotype is indirect. We note that GARS is essential for translation in all cells, and in neurons is active not only in the cell body, but also in the periphery of cells [33]. Local translation is essential for axon guidance, synaptic plasticity, cell migration, cell polarity and other areas of development and maintenance of the nervous system, and could be one phenomenon that in some way ameliorates the effect of the *SOD1^G93A^* mutation [34]–[36].

We note the intriguing finding of Kunst and colleagues that lysyl-tRNA synthetase (KARS) was one of only four proteins shown to interact with SOD1^G93A^ and SOD1^G85R^ in a yeast interaction trap experiment to find novel protein interactions with mutant SOD1 [37]. Recently, Kawamata and colleagues have shown that in mammalian cells mutant SOD1 interacts preferentially with the mitochondrial form of KARS [28]. These authors have also shown KARS-SOD1 interactions occur in the mitochondria of the nervous system in transgenic mice. In the presence of mutant SOD1, the mitochondrial form of KARS has a high propensity to aggregate prior to its import into mitochondria, becoming a target for proteasome degradation, and resulting in mitochondrial dysfunction [28]. Clearly it is now of interest to look at the mitochondrial function of GARS in light of these experiments.

Analysis of double heterozygous progeny from the *Dync1h1^Loa/+^* x *Gars^C201R/^* ^+^ cross showed a significantly more pronounced phenotype in terms of gait analysis and tremors than either parental strain, both of which have mild phenotypes. It is difficult to tell if we are seeing anything other than additive effects in these double heterozygote mice, but we note that for GARS to be involved in local translation, and possibly other non-canonical functions in the periphery of neurons, it must be anterogradely transported. Neurons maintain a balance of transport rates and flux in the anterograde and retrograde direction. Both *SOD1^G93A^* transgenics and *Dync1h1^Loa/+^* mice have deficits in axonal transport and this is thus another phenomenon that might affect GARS function [22], [26].

Our results show the *Gars^C201R/^* ^+^ mouse is an important addition to the range of mouse models available for studying neurodegenerative disease, both in directly modeling the human GARS mutation phenotypes and for teasing out the molecular interactions leading to pathogenesis in other neurodegenerative disorders. We note this mouse strain is freely available for research and we encourage its use.

## Materials and Methods

### Mice

The *SOD1^G93A^* parents for the cross described came from a colony in which the transgene array is maintained by crossing *SOD1^G93A^* hemizygous males to wildtype F1(SJL x C57BL/6) females, as recommended by the Jackson Laboratory (hemizygous *SOD1^G93A^* females are infertile).

*Dync1h1^Loa/+^* mice are congenic and are maintained by backcrossing to C57BL/6J mice. These mice are named according to the updated nomenclature for cytoplasmic dynein subunits [38], [39].

The *Gars^C201R^* mutation arose during an ENU mutagenesis experiment at the MRC Mammalian Genetics Unit, MRC Harwell, UK [40] and were assessed in a full SHIRPA test [24], [41] and other tests for locomotion; we positionally cloned the mutation and identified a T to C transition at base pair 456 that results in a non-conservative cysteine to arginine substitution at residue 201, as described [25]. All mice for this study were backcrossed to C57BL/6J.

Genotyping was performed at ∼40 days of age and all phenotypic characterization was performed blind to genotype, which was then decoded. The animal studies reported in this paper were carried out under the guidance issued by the Medical Research Council in *Responsibility in the Use of Animals for Medical Research* (1993) and under licence from the UK Home Office.

All mice were weighed at least once a week. However, when the *SOD1^G93A^* and *SOD1^G93A^*;*Gars^C201R/+^* mice started to show hindlimb weakness, these mice were weighed at least twice a week.

### Genotyping SOD1^G93A^, Dync1h1^Loa^ and Gars^C201R^ alleles

All mice were identified by genotyping for the presence of the human *SOD1* transgene array, and/or the *Dync1h1^Loa/+^* mutation and/or the *Gars^C201R^* mutation. *SOD1^G93A^* genotyping is as described previously [26] as is *Dync1h1^Loa^* genotyping [22]. The *Gars^C201R^* mutation introduces a restriction site for the enzymes HaeII and HhaI, allowing us to genotype by PCR followed by RFLP analysis. *Gars^C201R^* PCR primers (forward: CACGTGCTTGCTCTAGCAAGA; reverse: GTCTACCACTGAACACAGTCC) lying within intron 4 and intron 5 respectively, (spanning exon 5 of *Gars*) were used to amplify a 420 bp product. This amplicon is digested with HhaI to give fragments of 420 bp (no restriction site, wildtype *Gars*) and of 250 bp and 170 bp (*Gars^C201R^* mutant allele) [25].

### Grip strength testing

The grip strength test assessed neuromuscular function by measuring, with an electronic digital force gauge, the peak amount of force an animal applied in grasping a 10 cm×8 cm wire grid attached to a pull bar (Bioseb Instruments). The mouse was placed on the flat wire grid connected to the force gauge and held on with front and hind paws. It was held by the base of the tail and was gently pulled away from the grid until the mouse released its grip at which point peak tension on the pull bar was recorded. The mean of 5 measurements was determined for each mouse on each day of testing and the result normalized by weight. Further details of the Standard Operating Procedure for grip strength that we followed can be found at the Eumorphia site http://www.eumorphia.org/EMPReSS/servlet/EMPReSS.Frameset.

### Mouse wire walk test

Wildtype, *Dync1h1^Loa/+^* heterozygous, *Gars^C201R/+^* heterozygous and *Gars^C201R/+^*; *Dync1h1^Loa/+^* double heterozygous littermates, were tested at 7 months of age. Each animal was timed for one minute as it walked on the wire grid top of the cage, which has square holes (formed by the intercrossing wires) of 9 mm×80 mm. Mice were scored for every occasion in which they placed their foot in the hole rather than on a wire (‘foot placing error’).

### Assessment of muscle force and motor unit number

The maximum twitch and tetanic force of the tibialis anterior (TA) and extensor digitorum longus (EDL) muscles were assessed in littermates at either 120 days of age (*SOD1^G93A^*x*Gars^C201R/+^* cross) or 9 months of age (*Dync1h1^Loa/+^* x *Gars^C201R/^* ^+^ cross) as described in Kieran et al, 2005.

The animals were anaesthetized (4.5% chloral hydrate solution, 1 ml/100 g body weight, i.p.; Sigma-Aldrich, Poole, UK) and prepared for isometric tension recordings of muscle contraction [42]. The distal tendons of the TA and EDL muscles were exposed, dissected free from surrounding tissue and cut. The hind limbs of the animals were then rigidly secured to the table with stainless steel pins, and the distal tendons of the TA and EDL muscles attached to an isometric force transducer (Dynamometer UFI Devices) via silk thread. The sciatic nerve was exposed and sectioned, and all branches cut except for the deep peroneal nerve that innervates the TA and EDL muscles. The length of the muscles was adjusted for maximum twitch tension. The muscles and nerves were kept moist with saline throughout the recordings and all experiments were carried out at room temperature (23°C). Isometric contractions were elicited by stimulating the nerve to TA and EDL using square-wave pulses of 0.02-ms duration and supramaximal intensity via platinum electrodes. Tetanic contractions were elicited by trains of stimuli at a frequency of 40, 80, and 100 Hz. Maximum twitch and tetanic tension, time to peak, and half-relaxation time values were measured using a computer and appropriate software (Scope). The number of motor units in both EDL muscles was assessed by applying stimuli of increasing intensity to the motor nerve, resulting in stepwise increments in twitch tension, due to successive recruitment of motor axons. The number of stepwise increments was counted to give an estimate of the number of functional motor units present in each muscle.

#### Fatigue test

At the end of the isometric tension recordings, the fatigue pattern of the EDL muscles was assessed by repeatedly stimulating the muscle at 40 Hz for 250 ms every second for 3 mins, and the contractions were recorded on a pen recorder (Lectromed multitrace 2, UK Ltd). EDL is normally a fast fatiguable muscle that fatigues rapidly when repeatedly stimulated. From each fatigue trace, the decrease in tension after 3 min of stimulation was measured and a fatigue index (F.I.) was calculated: (initial tetanic tension – tetanic tension after stimulation)/initial tetanic tension.

#### Muscle weight, histochemistry and morphometry

At the end of each *in vivo* physiology experiment, the tibialis anterior (TA) and extensor digitorum longus (EDL) muscles (C57BL/6 background) were removed, weighed, and snap frozen in isopentane cooled in liquid nitrogen and stored at −80°C until processing. Serial cross sections (10 µm) of TA muscles were cut on a cryostat and stained for succinate dehydrogenase (SDH) activity to determine the oxidative capacity of the muscle fibers, as described previously [42]. The muscle sections were examined under a light microscope (Leica DMR) using Leica HC PL Fluotar objectives (10×, 20× and 40× magnification). The cross-sectional areas of the muscle fibers of from animals of each genotype were calculated from SDH-stained muscle sections using three sections from the belly of TA muscles from each mouse (*n = 3*). For each muscle section, the cross-sectional areas (CSA) of ∼2000 (+/−87) muscle fibers (approximately 70% of the TA muscle) were calculated by tracing around the fiber perimeters using Leica software. The analysis of CSA of muscle fibers is more accurate in exposing changes in fiber size than measuring other parameters such as muscle fiber diameter (Gorio et al., 1983).

#### Motor neuron survival

Following removal of the hindlimb muscles the mice were perfused transcardially with 4% PFA in 0.1 M phosphate buffer saline. The lumbar region of the spinal cord was removed, post-fixed in 4% PFA for 6 hours and cryoprotected in 30% sucrose for a minimum of 8 hours. Serial transverse sections (20 µm) were cut using a cryostat and stained with gallocyanin, a Nissl stain. Spinal cord sections were examined under a light microscope (Leica DMR) using Leica HC PL Fluotar objectives (10×, 20× and 40× magnification). The number of Nissl-stained motor neurons in the sciatic motor pool of every third section (n = 60) between the L2 and L5 levels of the spinal cord were counted. Only large (diameter>12 µm), polygonal neurons with a distinguishable nucleus and nucleolus and clearly identifiable Nissl structure were included in the counts. Images were captured using a Nikon E995 digital camera and the images downloaded into Adobe Photoshop CS. To optimise image contrast, Levels Adjustment operations were performed, but no other image manipulations were made.

#### Statistical analysis for muscle and motor neuron studies

Statistical significance among the groups was assessed using a Mann-Whitney *U* test, student t-test and ANOVA. Significance was set at *P*<0.05

### Western hybridization and protein quantification

Mice were killed according to UK Home Office regulations and brains and spinal cords were removed and flash frozen in liquid nitrogen. Tissue was homogenized in PBS with protease inhibitors (10% w/v) and the homogenate centrifuged for 20 min at 12,000 rpm at 4°C on a Beckman Coulter Allegra 25R centrifuge (TS-5.1–500 rotor) to remove cellular debris. The protein concentration of each sample was determined using a bicinchoninic acid assay (Pierce). Homogenates were electrophoresed on Polyacrylamide–Tris gels (16% or 4–20% gradient) and then transferred on to PVDF membrane (ImmobilonP). Membranes were washed for 1 hour in blocking solution (5% w/v skim milk powder, 0.05% Tween 20 in PBS), before addition of the primary antibody in blocking solution at 4°C overnight. The primary antibody was detected using AP conjugated anti-rabbit (Sigma) and the results visualised using CPD-Star (Roche). The blots were then stripped and reprobed using an antibody against β-actin (Sigma A5441). Autoradiographs were quantified using Imagemaster 1D software (Amersham Pharmacia Biotech) and the protein levels normalised for β-actin levels.

### Immunoprecipitation

Tissue homogenates were prepared according to the western hybridization protocol described above. Trublot anti-rabbit or anti-mouse Ig IP beads (eBioscience) were added to the homogenate and the suspension incubated for 1 hour at 4°C on a rotating shaker. The suspension was centrifuged to remove the beads and the primary antibody and fresh beads were added to the homogenate. The samples were incubated overnight at 4°C on a rotating shaker. The beads were then collected by centrifugation and washed three times in PBS with protease inhibitors and once in PBS with 0.05% Tween 20. The beads were collected by centrifugation and bound proteins removed by the addition of SDS loading buffer. Samples were analyzed by western blotting in accordance to the protocol above.

### Antibodies

The following antibodies were used in western blotting and immunoprecipitation protocols above: rabbit anti-GARS ([25] from Dr. Kevin Talbot); mouse anti-SOD1 (Novocastra); mouse anti-cytoplasmic dynein intermediate chain (Chemicon international); rabbit anti-dynein light chain LC8 (Abcam); goat anti-mouse IgG and goat anti-rabbit IgG alkaline phosphatase conjugated secondary antibodies (Sigma).

### Statistical analysis for muscle and motor neuron studies

Statistical significance among the groups was assessed using a Mann-Whitney *U* test, student t-test and ANOVA. Significance was set at *P*<0.05

## Supporting Information

Figure S1 EDL Muscle force in littermates from the SOD1G93A x GarsC201R/+ cross at 120 days of age. The bar charts show (A) the maximum twitch force and (B) maximum tetanic force generated by EDL muscles in littermates of each genotype. n = 5 female littermates per genotype. See Supplementary Table 1 for data. Error bars represent SEM.
(0.17 MB TIF)

Figure S2 GARS protein levels in *SOD1^G93A^* x *Gars^C201R/+^* and *Dync1h1^Loa/+^* x *Gars^C201R/+^* crosses at 4 months of age. (A) Representative western blot of GARS using spinal cord homogenates from progeny of the *SOD1^G93A^* x *Gars^C201R/+^* cross. β-actin blots are shown as loading controls. (B) Quantification of GARS protein levels in the progeny of the *SOD1^G93A^* x *Gars^C201R/+^* cross show a significant increase in GARS levels in *Gars^C201R/+^* and *SOD1^G93A^*;*Gars^C201R/+^* animals. Quantifications were normalized to β-actin. (C) Representative western blot of GARS using spinal cord homogenates from progeny of the *Dync1h1^Loa/+^* x *Gars^C201R/+^* cross. β-actin blots are shown as loading controls. (D) Quantification of GARS protein levels in the progeny of the *Dync1h1^Loa/+^* x *Gars^C201R/+^* cross show a significant increase in GARS levels in *Gars^C201R/+^* and *Dync1h1^Loa/+^*;*Gars^C201R/+^* animals. Quantifications were normalized to β-actin.
(0.38 MB TIF)

Figure S3 Four paw grip strength of sex-matched wildtype, *Dync1h1^Loa/+^*, *Gars^C201R/+^*, and *Dync1h1^Loa/+^*;*Gars^C201R/+^* littermates at 7 months of age, normalized by weight.
(0.23 MB TIF)

Figure S4 TA and EDL muscle weight and force and EDL motor unit survival and fatigue characterstics in littermates from the *Dync1h1^Loa/+^* x *Gars^C201R/+^* cross at 120 days of age. The bar charts show (A) the mean TA muscle weight and (B) the maximum tetanic force of TA; (C) the mean EDL muscle weight and (D) maximum tetanic force of EDL; (E) the mean number of motor units in the EDL muscle and (F) the mean fatigue index of EDL muscles. An FI approaching 1 indicates that the muscle is highly fatiguable. N = 3 for all genotypes; error bars are S.E.M
(0.48 MB DOC)

Table S1 Mean muscle force and weight of EDL muscles of littermates from the SOD1G93A x GarsC201R/+ cross at 120 days of age.
(0.03 MB DOC)

Table S2 Contractile and fatigue characteristics of EDL muscles of littermates from the *SOD1^G93A^* x *Gars^C201R/+^* cross at 120 days of age
(0.03 MB DOC)

Table S3 Mean muscle force and weight of TA and EDL muscles of littermates from the *Dync1h1^Loa/+^* x *Gars^C201R/+^* cross at 9 months of age.
(0.03 MB DOC)

Movie S1 Littermates from the *Dync1h1^Loa/+^* x *Gars^C201R/+^* cross at 6 months of age. Wildtype littermate.
(21.47 MB MPG)

Movie S2 Littermates from the *Dync1h1^Loa/+^* x *Gars^C201R/+^* cross at 6 months of age. *Dync1h1^Loa/+^* littermate.
(47.66 MB MPG)

Movie S3 Littermates from the *Dync1h1^Loa/+^* x *Gars^C201R/+^* cross at 6 months of age. *Gars^C201R/+^* littermate.
(47.05 MB MPG)

Movie S4 Littermates from the *Dync1h1^Loa/+^* x *Gars^C201R/+^* cross at 6 months of age. *Dync1h1^Loa/+^*;*Gars^C201R/+^* double heterozygote littermate.
(48.04 MB MPG)

## References

[1] Skre H (1974). Genetic and clinical aspects of Charcot-Marie-Tooth's disease.. Clin Genet.

[2] Banks GT, Kuta A, Isaacs AM, Fisher EM (2008). TDP-43 is a culprit in human neurodegeneration, and not just an innocent bystander.. Mamm Genome epub.

[3] The Amyotrophic Lateral Sclerosis Association (2002). http://www.alsa.org.

[4] Deng HX, Hentati A, Tainer JA, Iqbal Z, Cayabyab A (1993). Amyotrophic lateral sclerosis and structural defects in Cu,Zn superoxide dismutase.. Science.

[5] Rosen DR, Siddique T, Patterson D, Figlewicz DA, Sapp P (1993). Mutations in Cu/Zn superoxide dismutase gene are associated with familial amyotrophic lateral sclerosis.. Nature.

[6] James PA, Talbot K (2006). The molecular genetics of non-ALS motor neuron diseases.. Biochim Biophys Acta.

[7] Monani UR, Coovert DD, Burghes AH (2000). Animal models of spinal muscular atrophy.. Hum Mol Genet.

[8] Parman Y (2007). Hereditary neuropathies.. Curr Opin Neurol.

[9] Schymick JC, Talbot K, Traynor BJ (2007). Genetics of sporadic amyotrophic lateral sclerosis.. Hum Mol Genet.

[10] Sumner CJ (2007). Molecular mechanisms of spinal muscular atrophy.. J Child Neurol.

[11] Valdmanis PN, Rouleau GA (2008). Genetics of familial amyotrophic lateral sclerosis.. Neurology.

[12] Van Den BL, Timmerman V (2006). Genetics of motor neuron disease.. Curr Neurol Neurosci Rep.

[13] Puls I, Jonnakuty C, LaMonte BH, Holzbaur EL, Tokito M (2003). Mutant dynactin in motor neuron disease.. Nat Genet.

[14] Antonellis A, Ellsworth RE, Sambuughin N, Puls I, Abel A (2003). Glycyl tRNA synthetase mutations in Charcot-Marie-Tooth disease type 2D and distal spinal muscular atrophy type V.. Am J Hum Genet.

[15] Del Bo R, Locatelli F, Corti S, Scarlato M, Ghezzi S (2006). Coexistence of CMT-2D and distal SMA-V phenotypes in an Italian family with a GARS gene mutation.. Neurology.

[16] Dubourg O, Azzedine H, Yaou RB, Pouget J, Barois A (2006). The G526R glycyl-tRNA synthetase gene mutation in distal hereditary motor neuropathy type V.. Neurology.

[17] James PA, Cader MZ, Muntoni F, Childs AM, Crow YJ, Talbot K (2006). Severe childhood SMA and axonal CMT due to anticodon binding domain mutations in the GARS gene.. Neurology.

[18] Sivakumar K, Kyriakides T, Puls I, Nicholson GA, Funalot B (2005). Phenotypic spectrum of disorders associated with glycyl-tRNA synthetase mutations.. Brain.

[19] Gurney ME, Pu H, Chiu AY, Dal Canto MC, Polchow CY, Alexander DD (1994). Motor neuron degeneration in mice that express a human Cu,Zn superoxide dismutase mutation.. Science.

[20] Banks GT, Fisher EM (2008). Cytoplasmic dynein could be key to understanding neurodegeneration.. Genome Biol.

[21] Chen XJ, Levedakou EN, Millen KJ, Wollmann RL, Soliven B, Popko B (2007). Proprioceptive sensory neuropathy in mice with a mutation in the cytoplasmic Dynein heavy chain 1 gene.. J Neurosci.

[22] Hafezparast M, Klocke R, Ruhrberg C, Marquardt A, Ahmad-Annuar A (2003). Mutations in dynein link motor neuron degeneration to defects in retrograde transport.. Science.

[23] Ilieva HS, Yamanaka K, Malkmus S, Kakinohana O, Yaksh T (2008). Mutant dynein (Loa) triggers proprioceptive axon loss that extends survival only in the SOD1 ALS model with highest motor neuron death.. Proc Natl Acad Sci U S A.

[24] Rogers DC, Peters J, Martin JE, Ball S, Nicholson SJ (2001). SHIRPA, a protocol for behavioral assessment: validation for longitudinal study of neurological dysfunction in mice.. Neurosci Lett.

[25] Achilli F, Bros-Facer V, Williams HP, Banks GT, AlQatari M (2009). A novel mouse model with a point mutation in glycyl-tRNA synthetase (*Gars*) has sensory and motor phenotypes and profoundly reduced enzyme activity in homozygotes.. Dis Mod Mech in press.

[26] Kieran D, Hafezparast M, Bohnert S, Dick JRT, Martin J (2005). A mutation in dynein rescues axonal transport defects and extends the lifespan of ALS mice.. J Cell Biol.

[27] Achilli F, Boyle S, Kieran D, Chia R, Hafezparast M (2005). The SOD1 transgene in the G93A mouse model of amyotrophic lateral sclerosis lies on distal mouse chromosome 12.. Amyotroph Lateral Scler Other Motor Neuron Disord.

[28] Kawamata H, Magrane J, Kunst C, King MP, Manfredi G (2008). Lysyl-tRNA Synthetase Is a Target for Mutant SOD1 Toxicity in Mitochondria.. J Biol Chem.

[29] Kunst CB, Messer L, Gordon J, Haines J, Patterson D (2000). Genetic mapping of a mouse modifier gene that can prevent ALS onset.. Genomics.

[30] Teuchert M, Fischer D, Schwalenstoecker B, Habisch HJ, Bockers TM, Ludolph AC (2006). A dynein mutation attenuates motor neuron degeneration in SOD1(G93A) mice.. Exp Neurol.

[31] Heiman-Patterson TD, Deitch JS, Blankenhorn EP, Erwin KL, Perreault MJ (2005). Background and gender effects on survival in the TgN(SOD1-G93A)1Gur mouse model of ALS.. J Neurol Sci.

[32] Wooley CM, Sher RB, Kale A, Frankel WN, Cox GA, Seburn KL (2005). Gait analysis detects early changes in transgenic SOD1(G93A) mice.. Muscle Nerve.

[33] Nangle LA, Motta CM, Schimmel P (2006). Global effects of mistranslation from an editing defect in mammalian cells.. Chem Biol.

[34] Giuditta A, Kaplan BB, van Minnen J, Alvarez J, Koenig E (2002). Axonal and presynaptic protein synthesis: new insights into the biology of the neuron.. Trends Neurosci.

[35] Jordanova A, Irobi J, Thomas FP, Van Dijck P, Meerschaert K (2006). Disrupted function and axonal distribution of mutant tyrosyl-tRNA synthetase in dominant intermediate Charcot-Marie-Tooth neuropathy.. Nat Genet.

[36] Lin AC, Holt CE (2007). Local translation and directional steering in axons.. EMBO J.

[37] Kunst CB, Mezey E, Brownstein MJ, Patterson D (1997). Mutations in SOD1 associated with amyotrophic lateral sclerosis cause novel protein interactions.. Nat Genet.

[38] Pfister KK, Fisher EM, Gibbons IR, Hays TS, Holzbaur EL (2005). Cytoplasmic dynein nomenclature.. J Cell Biol.

[39] Pfister KK, Shah PR, Hummerich H, Russ A, Cotton J (2006). Genetic analysis of the cytoplasmic dynein subunit families.. PLoS Genet.

[40] Nolan PM, Peters J, Strivens M, Rogers D, Hagan J (2000). A systematic, genome-wide, phenotype-driven mutagenesis programme for gene function studies in the mouse.. Nat Genet.

[41] Rogers DC, Fisher EM, Brown SD, Peters J, Hunter AJ, Martin JE (1997). Behavioral and functional analysis of mouse phenotype: SHIRPA, a proposed protocol for comprehensive phenotype assessment.. Mamm Genome.

[42] Kieran D, Greensmith L (2004). Inhibition of calpains, by treatment with leupeptin, improves motoneuron survival and muscle function in models of motoneuron degeneration.. Neuroscience.

