# Correction: Mutant Glycyl-tRNA Synthetase (Gars) Ameliorates SOD1^G93A^ Motor Neuron Degeneration Phenotype but Has Little Affect on Loa Dynein Heavy Chain Mutant Mice

**DOI:** 10.1371/annotation/255006a5-407c-4c01-8e61-3be7ee39aad8

**Published:** 2009-10-07

**Authors:** Gareth T. Banks, Virginie Bros-Facer, Hazel P. Williams, Ruth Chia, Francesca Achilli, J. Barney Bryson, Linda Greensmith, Elizabeth M. C. Fisher

Table S1 is a duplicate of Table S2. Please view the correct Table S1 here: 
