# Supplementary figures and images for: Mutant Glycyl-tRNA Synthetase (Gars) Ameliorates SOD1G93A Motor Neuron Degeneration Phenotype but Has Little Affect on Loa Dynein Heavy Chain Mutant Mice
Source: PLoS One. 2009 Jul 13;4(7):e6218. doi: 10.1371/journal.pone.0006218 (PMC2704870; doi:10.1371/journal.pone.0006218)

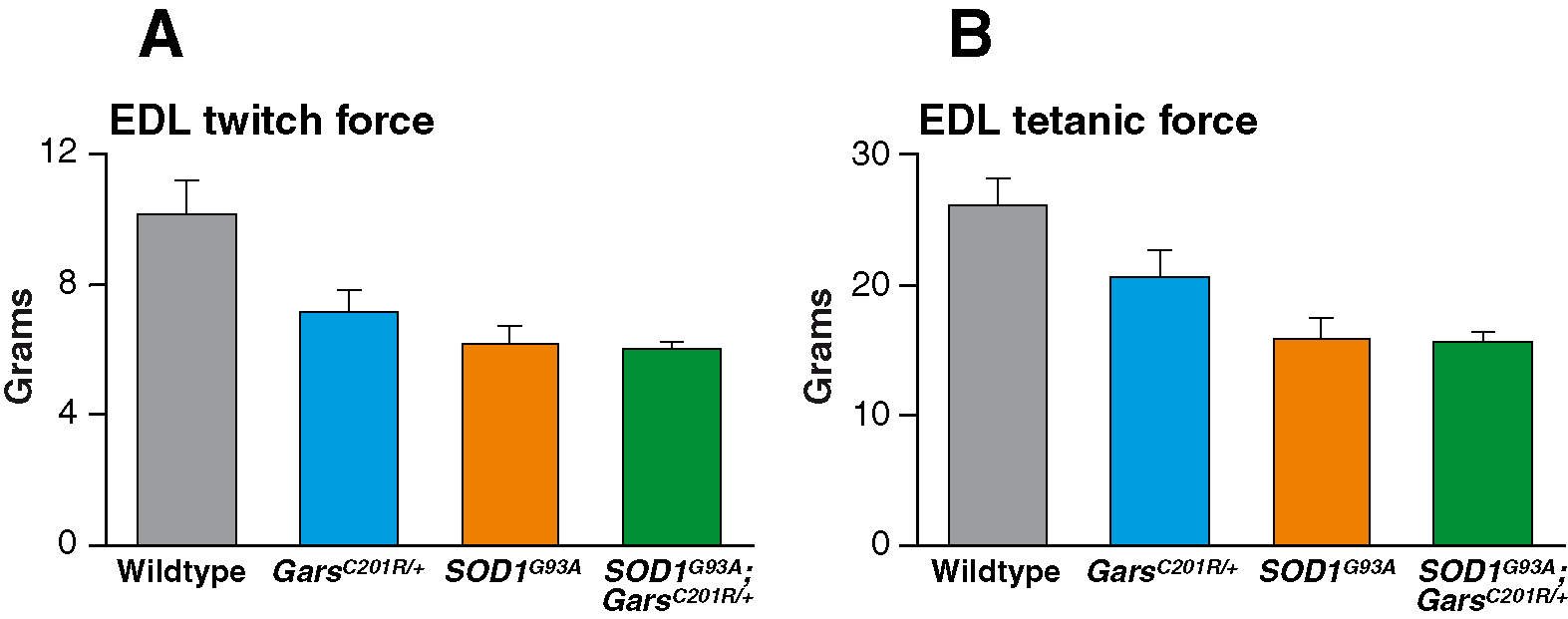

Supplement: Figure S1 — EDL Muscle force in littermates from the SOD1G93A x GarsC201R/+ cross at 120 days of age. The bar charts show (A) the maximum twitch force and (B) maximum tetanic force generated by EDL muscles in littermates of each genotype. n = 5 female littermates per genotype. See Supplementary Table 1 for data. Error bars represent SEM. (0.17 MB TIF) [file pone.0006218.s001.tif]

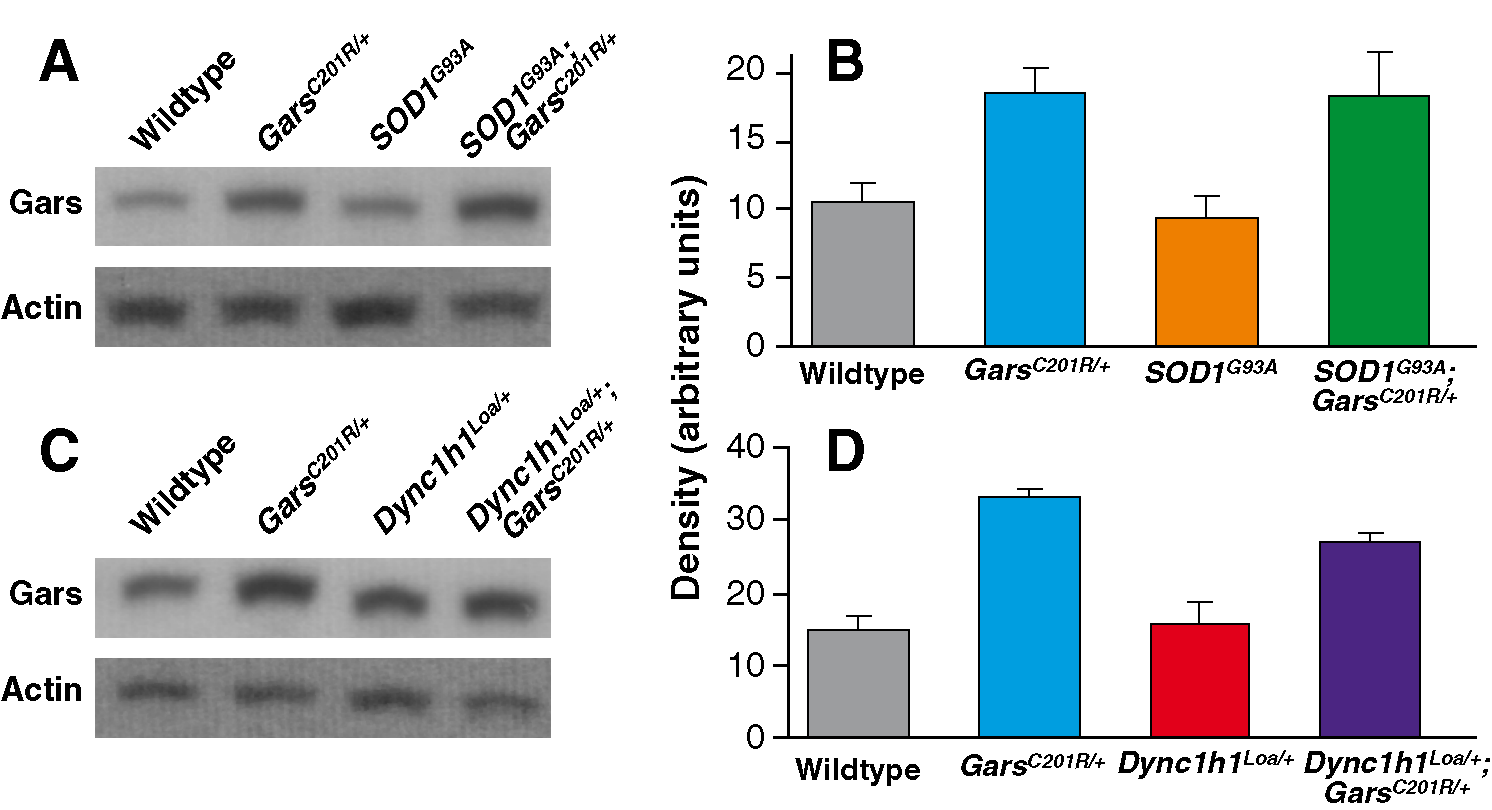

Supplement: Figure S2 — GARS protein levels in SOD1G93A x GarsC201R/+ and Dync1h1Loa/+ x GarsC201R/+ crosses at 4 months of age. (A) Representative western blot of GARS using spinal cord homogenates from progeny of the SOD1G93A x GarsC201R/+ cross. β-actin blots are shown as loading controls. (B) Quantification of GARS protein levels in the progeny of the SOD1G93A x GarsC201R/+ cross show a significant increase in GARS levels in GarsC201R/+ and SOD1G93A;GarsC201R/+ animals. Quantifications were normalized to β-actin. (C) Representative western blot of GARS using spinal cord homogenates from progeny of the Dync1h1Loa/+ x GarsC201R/+ cross. β-actin blots are shown as loading controls. (D) Quantification of GARS protein levels in the progeny of the Dync1h1Loa/+ x GarsC201R/+ cross show a significant increase in GARS levels in GarsC201R/+ and Dync1h1Loa/+;GarsC201R/+ animals. Quantifications were normalized to β-actin. (0.38 MB TIF) [file pone.0006218.s002.tif]

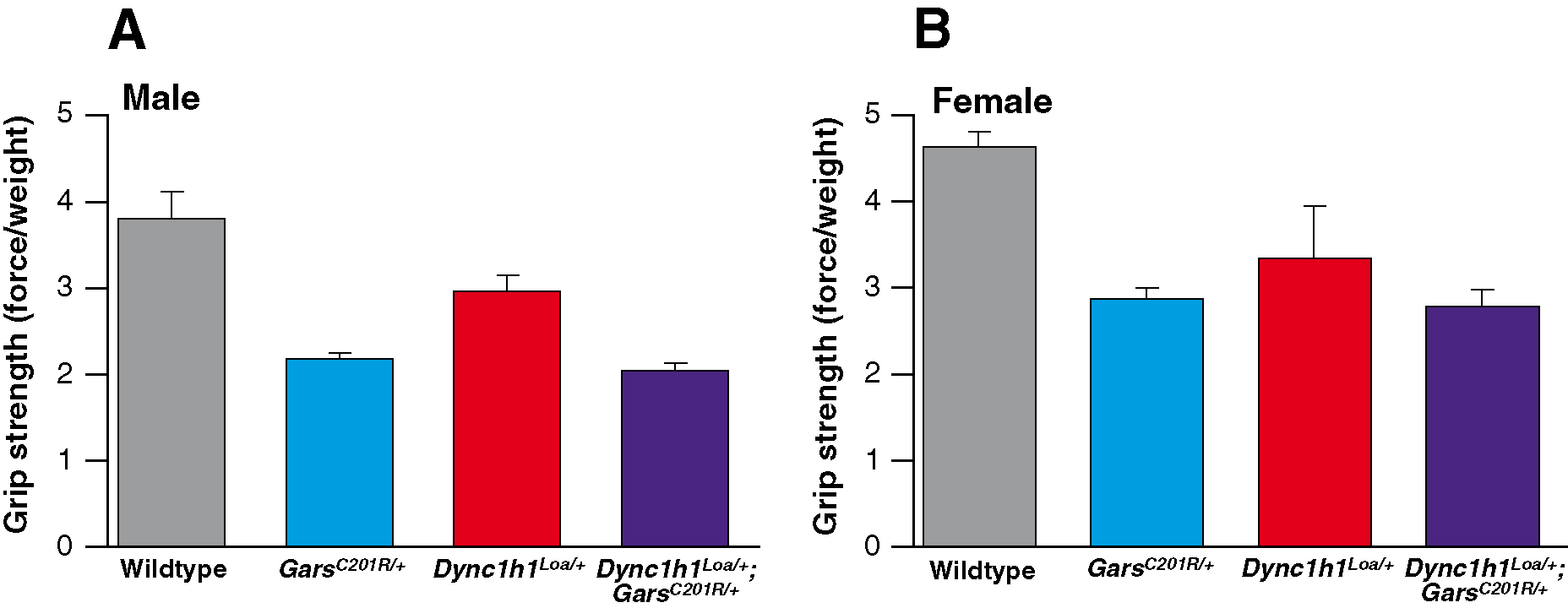

Supplement: Figure S3 — Four paw grip strength of sex-matched wildtype, Dync1h1Loa/+, GarsC201R/+, and Dync1h1Loa/+;GarsC201R/+ littermates at 7 months of age, normalized by weight. (0.23 MB TIF) [file pone.0006218.s003.tif]

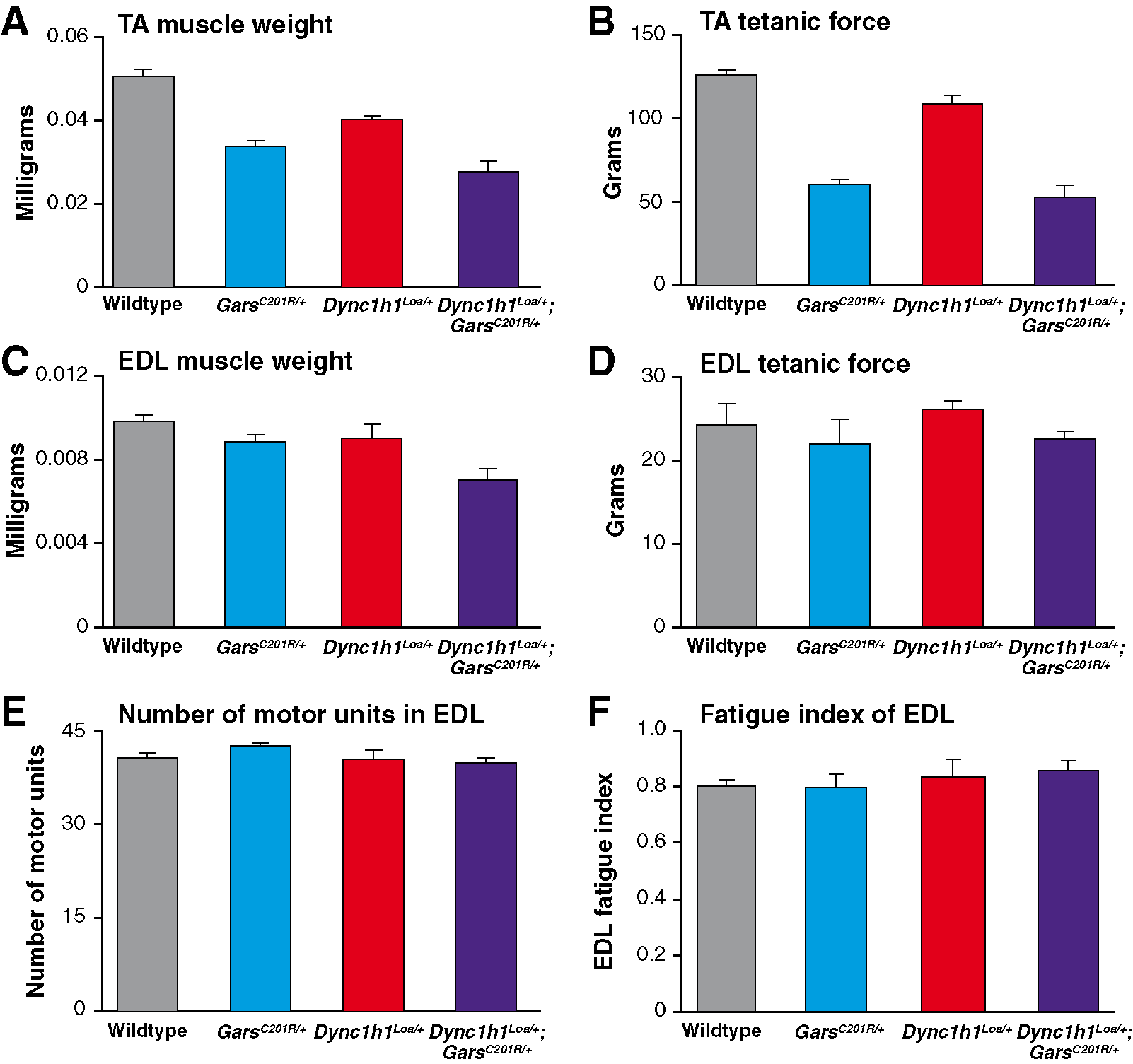

Supplement: Figure S4 — TA and EDL muscle weight and force and EDL motor unit survival and fatigue characterstics in littermates from the Dync1h1Loa/+ x GarsC201R/+ cross at 120 days of age. The bar charts show (A) the mean TA muscle weight and (B) the maximum tetanic force of TA; (C) the mean EDL muscle weight and (D) maximum tetanic force of EDL; (E) the mean number of motor units in the EDL muscle and (F) the mean fatigue index of EDL muscles. An FI approaching 1 indicates that the muscle is highly fatiguable. N = 3 for all genotypes; error bars are S.E.M (0.48 MB DOC) [file pone.0006218.s004.doc]
