# Supplementary material for: Mutant Glycyl-tRNA Synthetase (Gars) Ameliorates SOD1G93A Motor Neuron Degeneration Phenotype but Has Little Affect on Loa Dynein Heavy Chain Mutant Mice
Source: PLoS One. 2009 Jul 13;4(7):e6218. doi: 10.1371/journal.pone.0006218 (PMC2704870; doi:10.1371/journal.pone.0006218)
Supplement: Table S1 — Mean muscle force and weight of EDL muscles of littermates from the SOD1G93A x GarsC201R/+ cross at 120 days of age. (0.03 MB DOC) [file pone.0006218.s005.doc]

**Supplementary Table 2. Contractile and fatigue characteristics of EDL muscles of littermates from the *SOD1^G93A^* x *Gars^C201R/^*^+^ cross** **at 120 days of age**

All mice were female and n=5 for all genotypes. Values are ± SEM.

| Genotype | Time to Peak (TTP) | Half Relaxation Time (1/2 RT) | Fatigue Index |
| --- | --- | --- | --- |
| Wildtype | 29.96±1.12 | 47.74±1.48 | 0.69±-0.03 |
| SOD^G93A^ | 32.43±0.79 | 66.53±2.31 | 0.69±0.04 |
| *Gars^C201R^*^/+^ | 30.02±1.42 | 51.40±1.77 | 0.82±0.20 |
| *SOD1^G93A^*;*Gars^C201R/+^* | 30.81±1.63 | 60.22±4.21 | 0.72±0.04 |
