# Supplementary material for: Mutant Glycyl-tRNA Synthetase (Gars) Ameliorates SOD1G93A Motor Neuron Degeneration Phenotype but Has Little Affect on Loa Dynein Heavy Chain Mutant Mice
Source: PLoS One. 2009 Jul 13;4(7):e6218. doi: 10.1371/journal.pone.0006218 (PMC2704870; doi:10.1371/journal.pone.0006218)
Supplement: Table S3 — Mean muscle force and weight of TA and EDL muscles of littermates from the Dync1h1Loa/+ x GarsC201R/+ cross at 9 months of age. (0.03 MB DOC) [file pone.0006218.s007.doc]

**Supplementary Table 3. Mean muscle force and weight of TA and EDL muscles of littermates from the *Dync1h1^Loa/+^x Gars^C201R/+^* cross at 9 months of age.**

All mice were female and n=3 for all genotypes (except *Dync1h1^Loa/+^* where n=2). Values ± SEM.

| Genotype | TA Tetanus force (g) | EDL Tetanus values (g) | TA muscle weights (mg) | EDL muscle weights (mg) |
| --- | --- | --- | --- | --- |
| Wildtype | 126.1 ± 2.7 | 40.6 ± 0.7 | 50.6 ±1.7 | 9.8±0.30 |
| *Dync1h1^Loa/+^* | 109.0 ±5.1 | 40.3 ±1.5 | 40.3 ±0.9 | 9.0 ±0.70 |
| *Gars^C201R^*^/+^ | 60.6 ±3.0 | 42.5 ±0.4 | 33.8 ± 1.2 | 8.9 ±0.34 |
| *Dync1h1^Loa/+^;Gars^C201R/+^* | 52.6 ±7.3 | 39.8 ±0.9 | 27.7 ±2.5 | 7.0 ±0.30 |
