# Supplementary figures and images for: Correction: Mutant Glycyl-tRNA Synthetase (Gars) Ameliorates SOD1G93A Motor Neuron Degeneration Phenotype but Has Little Affect on Loa Dynein Heavy Chain Mutant Mice
Source: PLoS One. 2009 Oct 7;4(10):10.1371/annotation/255006a5-407c-4c01-8e61-3be7ee39aad8. doi: 10.1371/annotation/255006a5-407c-4c01-8e61-3be7ee39aad8 (PMC2763892; doi:10.1371/annotation/255006a5-407c-4c01-8e61-3be7ee39aad8)

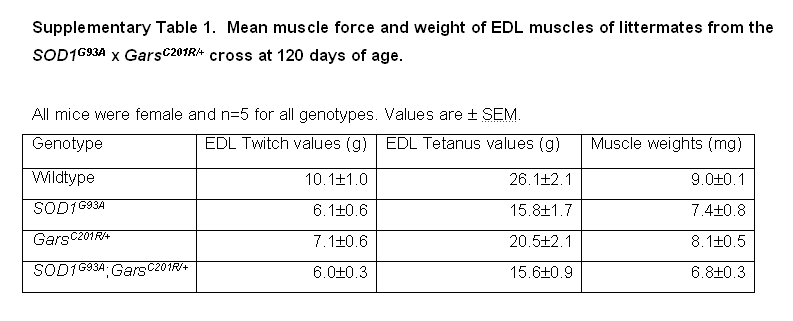

Supplement: Supplementary file 1 [file pone.255006a5-407c-4c01-8e61-3be7ee39aad8.s001.tif]
